# Synergistic Combination of Letrozole and Berberine in Ascorbic Acid-Stabilized AuNPs: A Promising Solution for Breast Cancer

**DOI:** 10.3390/ph16081099

**Published:** 2023-08-03

**Authors:** Ahmed I. Foudah, Aftab Alam, Mohammad Ayman Salkini, Samir A. Ross, Piyush Kumar, Mohammed F. Aldawsari, Mohammed H. Alqarni, Sherouk Hussein Sweilam

**Affiliations:** 1 Department of Pharmacognosy, College of Pharmacy, Prince Sattam Bin Abdulaziz University, Alkharj 11942, Saudi Arabia; a.alam@psau.edu.sa (A.A.); m.salkini@psau.edu.sa (M.A.S.); m.alqarni@psau.edu.sa (M.H.A.); s.sweilam@psau.edu.sa (S.H.S.); 2 National Center for Natural Products Research, School of Pharmacy, The University of Mississippi, Oxford, MS 38677, USA; sross@olemiss.edu; 3 Department of Biomolecular Sciences, School of Pharmacy, The University of Mississippi, Oxford, MS 38677, USA; 4 Department of Chemistry, Indian Institute of Technology, NH-44, PO Nagrota, Jagti, Jammu 181221, India; piyus.kumar@iitjammu.ac.in; 5 Department of Pharmaceutics, College of Pharmacy, Prince Sattam Bin Abdulaziz University, Alkharj 11942, Saudi Arabia; moh.aldawsari@psau.edu.sa; 6 Department of Pharmacognosy, Faculty of Pharmacy, Egyptian Russian University, Cairo-Suez Road, Badr City, Cairo 11829, Egypt

**Keywords:** breast cancer, letrozole, berberine, nanoparticles, gold nanoparticles

## Abstract

Breast cancer is a deadly disease that affects countless women worldwide. The most conventional treatments for breast cancer, such as the administration of anticancer medications such as letrozole (LTZ), pose significant barriers due to the non-selective delivery and low bioavailability of cytotoxic drugs leading to serious adverse effects and multidrug resistance (MDR). Addressing these obstacles requires an innovative approach, and we propose a combined strategy that synergistically incorporates LTZ with berberine (BBR) into stabilised AuNPs coated with ascorbic acid (AA), known as LTZ-BBR@AA-AuNPs. The LTZ-BBR@AA-AuNPs, a novel combined drug delivery system, were carefully designed to maximise the entrapment of both LTZ and BBR. The resulting spherical nanoparticles exhibited remarkable efficiency in trapping these two compounds, with rates of 58% and 54%, respectively. In particular, the average hydrodynamic diameter of these nanoparticles was determined to be 81.23 ± 4.0 nm with a PDI value of only 0.286, indicating excellent uniformity between them. Furthermore, their zeta potential was observed to be −14.5 mV, suggesting high stability even under physiological conditions. The release profiles showed that after being incubated for about 24 h at pH levels ranging from acidic (pH = 5) to basic (pH = 7), the percentage released for both drugs ranged from 56–72%. This sustained and controlled drug release can reduce any negative side effects while improving therapeutic efficacy when administered directly to cancer. MDA-MB-231 cells treated with LTZ-BBR@AA-AuNPs for 48 h exhibited IC_50_ values of 2.04 ± 0.011 μg/mL, indicating potent cytotoxicity against cells. Furthermore, the nanoparticles demonstrated excellent stability throughout the duration of the treatment.

## 1. Introduction

Breast cancer is the most common malignancy in women worldwide, and its prevalence is increasing year by year [1]. Breast cancer is caused by the malignant growth of epithelial cells that line the excretory ducts or lobules of the breast. Despite significant advances in early detection and treatment methods, breast cancer is still considered a leading cause of mortality in women [2]. Over many years, breast cancer has posed a significant threat to several generations. The number of breast cancer cases (18.1 million new cases) and deaths (9.6 million) is increasing every year, according to GLOBACON 2018. With an estimated incidence rate of 11.7% of people worldwide in 2020, breast cancer is the leading cause of cancer occurrence among all other cancers [3]. Estrogen levels moderate the growth and spread of breast tumours, making this cancer hormone-dependent [4].

Conventional breast cancer treatment involves surgery to remove tumours, radiation therapy to destroy remaining cancer cells, chemotherapy to target rapidly dividing cells, hormonal therapy for hormone receptor-positive cancers, and targeted therapy to block specific proteins or genes [5,6,7]. Ongoing research aims to improve treatment efficacy, minimize side effects, overcome drug resistance, and personalize treatments based on individual factors and tumour characteristics.

Most breast cancers are susceptible to hormonal stimulation, with estrogen activating estrogen receptors and playing a critical role in cancer development and progression [8]. Treatment of breast cancer at all stages can be effective by blocking estrogen synthesis or receptor activity [9]. Among the most successful therapies for hormone-dependent breast cancer is chemotherapy with cytotoxic drugs. Letrozole, or LTZ, is an example of one of these treatments. LTZ is an aromatase inhibitor that is often used to treat breast cancer that is estrogen receptor-positive. It is a potent aromatase inhibitor and is more effective in the treatment of postmenopausal breast cancer in hormone-sensitive women than other known selective estrogen receptor modulators such as tamoxifen [10]. It achieves this by preventing the conversion of testosterone to estradiol (a precursor of estrogen) [11]. 

The pharmacokinetics of most anticancer drugs vary widely from patient to patient, which can lead to inconsistent antitumour efficacy and unexpected toxicity [12]. On the other hand, the vast majority of patients do not respond favourably to these prescription drugs and often suffer serious adverse effects as a result of the fact that systemic administration of chemotherapeutic agents damages healthy cells in ways that do not selectively target cancer cells. This leads to a variety of adverse side effects [13]. The main reason is that treatment destroys both normal and tumour cells, and the concentration of the drug in tumour cells is insufficient. The main obstacles to the effectiveness of cancer chemotherapy are drug resistance and dose-limiting toxicity [14]. 

Chemotherapy can kill cancer cells and prevent tumour recurrence when administered correctly. However, the prolonged use of single-agent chemotherapy regimens often limits their therapeutic efficacy. Drug combination therapy is a promising strategy to enhance the desired therapeutic effect while reducing side effects [15,16,17]. Combining agents with different mechanisms of action was the first solution to the problem of resistance to chemotherapy with a single agent [18]. Chemotherapy combined with complementary and alternative herbal medicine has created a new paradigm of cancer treatment that has led to more comprehensive therapies [19]. Numerous case studies describe the successful integration of anticancer drugs and complementary therapies in the treatment of breast cancer. For example, Atallah et al. reported the efficacy of a chemo–plant combination therapy for the treatment of breast cancer that included pemetrexed and honokiol [20]. In another research article, Khan et al. found that the nanoparticulate carrier poly(lactic-co-glycolic acid) (PLGA) effectively delivered both doxorubicin and the naturally occurring bioactive anticancer compound berberine to cancer cells [21]. The synergistic character of drug molecules in cancer treatment offers the potential for improved therapeutic outcomes, reduced drug resistance, and enhanced patient well-being.

However, the severity of chemotherapy regimens has limited the use and benefits of chemotherapeutic agents. Alternative cancer treatments include herbal medicine, which is often used to treat the disease [22,23,24]. In addition, naturally occurring substances provide higher biological or pharmacological activities that are potentially beneficial for cancer treatment, better antitumour activity, and lower toxicity than synthetic substances [25]. In recent years, there has been a strong focus on the search for naturally occurring chemo-preventive polyherbal substances, especially those found in food and medicinal plants. Curcumin, quercetin, berberine, and other bioactive natural anticancer compounds have recently been used to treat various malignancies [21]. Remarkably, further research has shown that berberine, or BBR, is a natural alkaloid extracted from the rhizome of the Cupid plant, which is known to be an effective anticancer agent. It now exhibits various pharmacological effects, including antibacterial, anticholinergic, antihypertensive, anti-inflammatory and antioxidant properties [26].

Recent research has shown that nanoparticles loaded with anticancer drugs are a superior technology for the delivery of drugs with higher efficacy [1,27]. One technique is the use of metallic nanoparticles, representing a new approach to cancer treatment. In this method, gold nanoparticles (AuNPs) are injected and bound to tumour sites [28]. Due to their excellent compatibility with the biological system, small size, low toxicity, easy surface adaptation, and controllable drug release, AuNPs are among the inorganic nanoparticles most commonly used in biomedicine [29]. Because they have non-cell-damaging, biocompatible, immunologically safe, and adaptable surface functions, they can be used for drug delivery [30]. To enhance the stability of AuNPs, ascorbic acid was incorporated into the method. During the synthesis process, it functions as a stabilizer and plays a role in reducing AuNPs and protecting them from oxidation. The effectiveness and potential degradation or aggregation of AuNPs can be prevented while also enhancing their stability and integrity through the incorporation of ascorbic acid [31,32].

With the goal of reducing the toxicity associated with letrozole (LTZ), a novel approach was pursued. The strategy involved the combination of LTZ with berberine (BBR), a bioactive anticancer agent derived from plants. By doing so, the cytotoxic effects of LTZ could be mitigated. Additionally, instead of using conventional nanoparticles, metal nanocarriers were employed for sequential distribution of the compounds through conjugation and encapsulation. The investigation focused on evaluating the cytotoxicity of the nanocomplex containing LTZ and BBR in MDA-MB breast cancer cells at varying doses.

## 2. Results

### 2.1. Preparation and Fourier Transform Infrared Analysis of LTZ-BBR@AA-AuNPs

**Figure 1:**
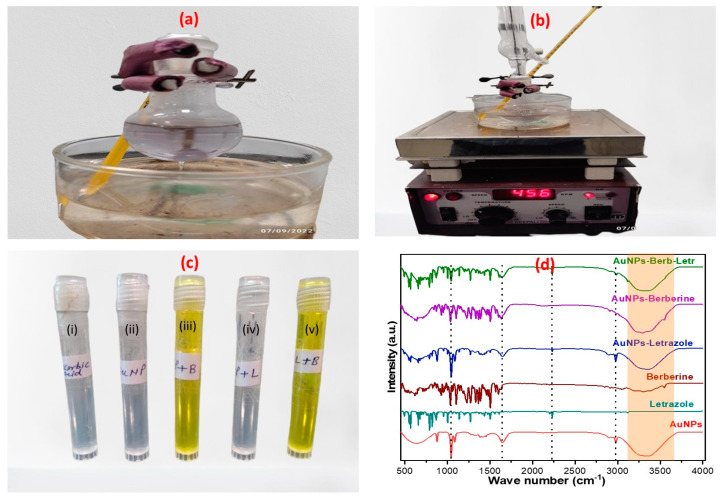
(**a**,**b**) Images depicting the various stages of formulation and development for LTZ-BBR@AA-AuNPs; (**c**) an image illustrating the final formulation of LTZ-BBR@AA-AuNPs, insert (i) ascorbic acid, (ii) AuNPs, (iii) BBR-AA-AuNPs, (iv) LTZ-@AA-AuNPs, (v) LTZ-BBR@AA-AuNPs; (**d**) FTIR spectra of AuNPS and their composition.

At room temperature, the LTZ-BBR@AA-AuNP mixture was a clear and transparent liquid with a distinct yellow hue, as shown in Figure 1. The characteristic spectra of berberine, letrozole, and ascorbic acid showed stretching of alkane groups (-CH,-CH_2_,-CH_3_) at 2800–3000 cm^−1^. The spectra of a solution of L-ascorbic acid showed that the stretching vibration of a C-C double bond was seen at 1645 cm^−1^, along with the peak of an enol hydroxyl at 1384 cm^−1^. The most represented berberine vibration modes were found at 1504 cm^−1^ (aromatic C=C vibrations), 1103 cm^−1^ (ring deformation and CH in-plane bending), and 1034 cm^−1^ (ring deformation and CH in-plane vibrations). The characteristic absorption peaks of letrozole were detected at 1270 cm^−1^ for C-N stretching and 2231 cm^−1^ for -CN stretching. The observed bands demonstrate that the gold nanoparticles contain both berberine and letrozole molecules close to each other.

### 2.2. Drug Capping

A UV spectrophotometer determined each drug’s presence in LTZ and BBR. The results confirmed that LTZ and BBR effectively encapsulated the drugs. LTZ and BBR achieved high drug-loading efficiencies of 58% and 54%, respectively.

### 2.3. Shape, Particle Size, Polydispersity Index, and Zeta Potential

**Figure 2:**
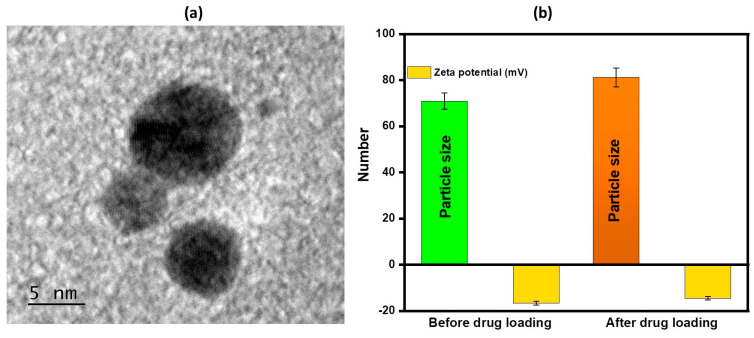
(**a**) TEM micrograph illustrating the LTZ-BBR@AA-AuNPs’ formulation; (**b**) particle size, polydispersity index (PDI), and zeta potential were assessed before and after drug loading.

The TEM image depicted a magnified view of the surface of a material called LTZ-BBR@AA-AuNP, which contained nanoparticles. The particles appeared nearly spherical, and their average size was approximately 5 nm, as clearly observed in Figure 2a. The size of particles and their zeta potential play a crucial role in maximizing the effectiveness of cancer treatment. These factors significantly impact the performance of the delivery system. In a study, it was observed that the average hydrodynamic diameters of AA-AuNPs and LTZ-BBR@AA-AuNPs were measured to be 70.89 ± 3.54 nm and 81.23 ± 4.0 nm, respectively. The polydispersity index (PDI) values for LTZ-BBR@AA-AuNPs and AA-AuNPs were 0.309 and 0.286, respectively. Additionally, the zeta potential of LTZ-BBR@AA-AuNPs was determined, and the results confirmed the successful formation of nanoparticles. AA-AuNPs and LTZ-BBR@AA-AuNPs exhibited zeta potentials of −16.6 mV and −14.5 mV, respectively, as depicted in Figure 2b.

### 2.4. In Vitro Drug Release Studies

**Figure 3:**
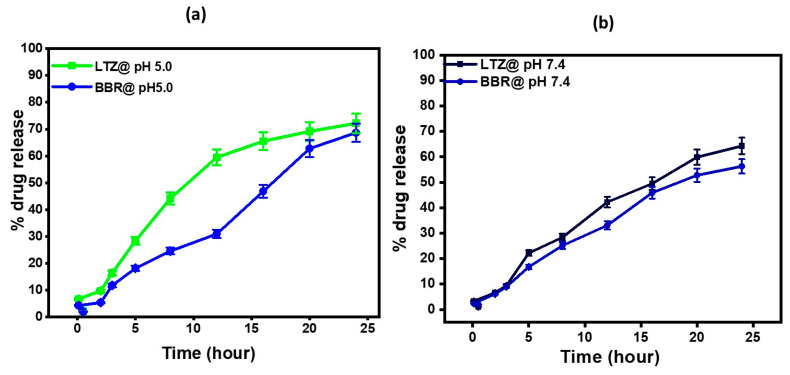
(**a**) In vitro drug release studies conducted at pH 5.0; (**b**) represents in vitro drug release studies conducted at pH 7.4 over a 24-h period.

To assess the release of the drug, each sample (2 mL) was placed in a dialysis bag made of semipermeable acetate cellulose and sealed. These bags were then submerged in 50 mL of the chosen release medium. The drug release study of LTZ-BBR@AA-AuNPs was conducted under two pH conditions: 5.0 and 7.4, representing the environment of cancer cells and physiological conditions, respectively. Figure 3a,b illustrate the release profiles of LTZ and BBR at the two pH values. At pH 5.0, after 3 h, the drug release percentages were 16.45 ± 0.82% for LTZ and 11.8 ± 0.59% for BBR. After 24 h, the release percentages increased to 72.18 ± 3.69% for LTZ and 68.7 ± 3.43% for BBR. At pH 7.4, the drug release percentages were 9.38 ± 0.469% for LTZ and 8.92 ± 0.446% for BBR after 3 h. After 24 h, the release percentages were 64.3 ± 3.215% for LTZ and 56.3 ± 2.815% for BBR. The sustained release of the nanoparticles is an important characteristic, as it maintains constant therapeutic concentrations of LTZ and BBR over an extended period. This sustained release is crucial for effective treatment. 

### 2.5. Anticancer Activity 

**Figure 4:**
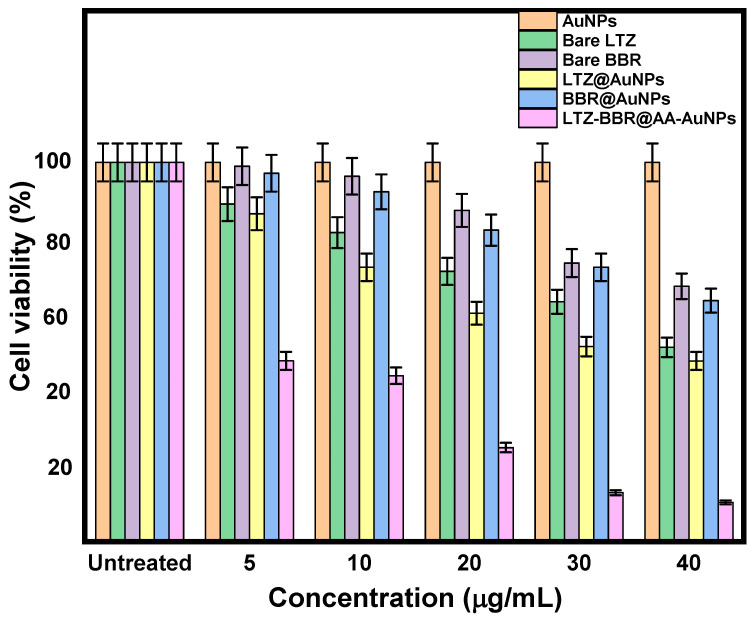
Effect of individual and combined AuNP (LTZ and BBR) treatment on MDA-MB-231 cell lines.

MDA-MB-231 cells underwent various treatments involving different concentrations ranging from 5–40 µg/mL. The treatments included naked LTZ, naked BBR, LTZ@AuNPs, BBR@AuNPs, and LTZ-BBR@AA-AuNPs, and they lasted for 48 h. The viability of the cells was evaluated using the MTT assay, as illustrated in Figure 4. In all treatment groups, there was an observed reduction in cell viability, which depended on both the dose and duration of treatment. Notably, the combined treatment of LTZ and BBR for 48 h resulted in significantly lower cell viability (33.7 ± 1.68%) compared to the groups treated with LTZ@AuNPs (47.62 ± 2.38%) and BBR@AuNPs (67.36 ± 3.36%) alone. Conversely, the percentages of cell viability for naked LTZ and naked BBR treatments were 51.23 ± 2.56% and 67.36 ± 3.36%, respectively. Moreover, the IC50 values for MDA-MB-231 cells treated with LTZ-BBR@AA-AuNPs after 48 h were determined to be 2.04 ± 0.011 μg/mL. These findings demonstrate the enhanced cytotoxicity and synergistic effect of the combined treatment using LTZ and BBR within the AA-AuNP formulation, highlighting its potential as an effective strategy for breast cancer treatment.

### 2.6. Estimation of Intracellular ROS by ELISA

**Figure 5:**
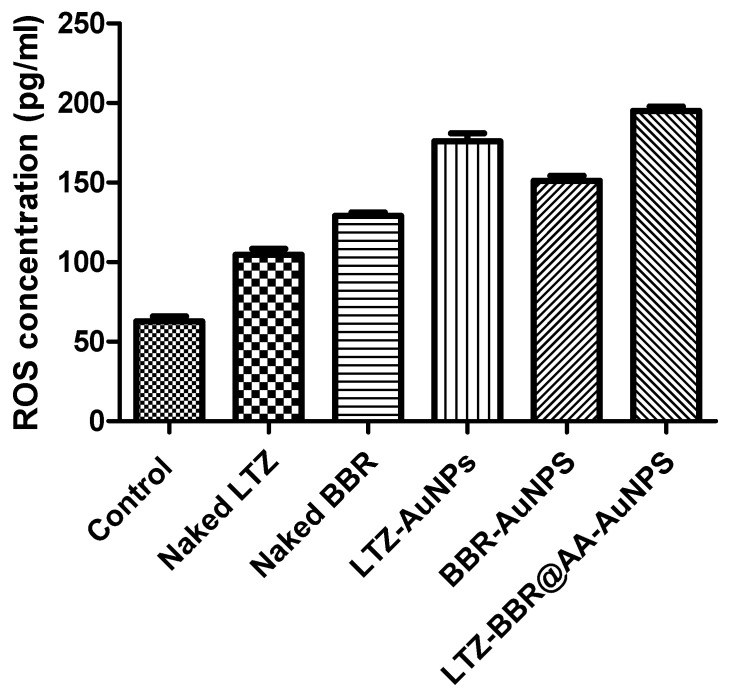
Estimation of intracellular ROS by ELISA. Values are expressed as mean ± SEM in each group. Significance was determined by a two-way analysis of variance. *p* < 0.001 when compared to the control group (*p* < 0.05).

To determine the levels of reactive oxygen species in MDA-MB-231 cells, ELISA assays were used. The results are shown in Figure 5. The research found that the control group had noticeably lower levels of ROS compared to all other treatment groups, which included naked LTZ and BBR, as well as various combinations using AuNPs such as LTZ@AuNPs, BBR@AuNPs, and combinational therapies of LTZ-BBR@AA-AuNPs. There was a fluctuation in the increase of ROS levels between various treatment groups, compared to the control. The increase in potency was evident in all formulations. LTZ use resulted in a 1.6-fold increase, while BBR showed a 2.01-fold increase when used to aid cellular uptake or protect against degradation/metabolism. LTZ@AuNPs showed a relatively modest 2.7-fold change, while BBR@AuNPs had an approximately 2-fold improvement compared to their naked counterparts (*p* < 0.05). However, optimal efficacy was achieved by combining LTZ and BBR with AA-AuNP conjugates, resulting in markedly enhanced performance displaying the greatest change at 3.06-fold (*p* < 0.05).

The rise in ROS levels seen among all the groups treated indicates that the given substances or nanoparticles caused oxidative stress within MDA-MB-231 cell types. The results suggest that the use of LTZ and BBR alone or in conjunction with AuNPs (LTZ@AuNPs and BBR@AuNPs) may disrupt the cellular redox balance, leading to increased production of reactive oxygen species. Furthermore, when LTZ-BBR@AA-AuNPs were administered in combination, the increase in ROS levels was significantly higher than those of each treatment alone or that of the control group. This finding suggests a possible synergistic action between these agents.

Understanding the impact of delivered substances or formulations on the cellular oxidative state is critical [33]. Elevated levels of reactive oxygen species can lead to various consequences, including damage to cellular components, modification of signalling pathways, and the initiation of cell death mechanisms such as apoptosis and necrosis [34,35]. The significance of the research lies in comprehending these results, as they help to develop new therapeutic approaches that improve conditions associated with oxidative stress while mitigating any adverse effects associated with increased ROS production.

### 2.7. Cell Cycle Analysis by Flow Cytometry

**Figure 6:**
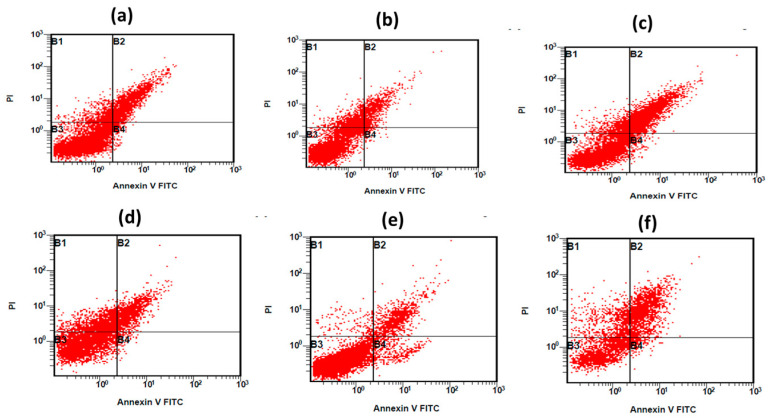
Annexin V-FITC/PI analysis of apoptosis in MDA-MB-231 breast cancer cells induced by (**a**) no treatment; (**b**) naked LTZ; (**c**) naked BBR; (**d**) LTZ@AuNPs; (**e**) BBR@AuNPs; (**f**) LTZ-BBR@AA-AuNPs.

To comprehensively assess the cytotoxic effect of naked LTZ, naked BBR, LTZ@AuNPs, BBR@AuNPs, and their combination of LTZ-BBR@AA-AuNPs (in DMSO) on MDA-MB-231 cell growth, we have utilised a highly sensitive flow cytometry technique in conjunction with annexin V/PI staining. To determine the optimal concentrations of treatments, extensive analysis was conducted on their IC_50_ values. Upon careful examination of the data obtained from flow cytometry analysis and annexin V/PI staining, a significant increase in apoptosis and necrosis was observed upon treatment with LTZ, BBR, LTZ@AuNPs, BBR@AuNPs, and their combination. These findings showed that these treatments have not only been effective but have also induced an encouraging outcome compared to untreated cells (Figure 6). In light of these results, it seems that the therapies administered had a notable effect in increasing programmed cell death within the MDA-MB-231 cell line. These findings provide valuable insights into the mechanism of action of the treatments and contribute to our understanding of their potential as anticancer agents.

### 2.8. Stability Study

We conducted a stability study on LTZ-BBR@AA-AuNPs to evaluate their colour, transparency, and efficiency of drug capping for BBR and LTZ over a period of 30 days, as reported in Table 1. Throughout the study, the nanoparticles’ colour remained yellow, indicating no substantial alterations or deterioration. The nanoparticles’ transparency was unaffected. During the investigation, BBR capping on nanoparticles showed no variance in drug-capping effectiveness. Initially, at day 0, the drug-capping efficiency for BBR was 54.33 ± 0.47%. After 15 days, it had slightly increased to 54.87 ± 0.17%, and by the end of the 30-day period, it had reached 56.14 ± 0.65%. These findings suggest that the drug capping of BBR on the LTZ-BBR@AA-AuNP formulation remained stable throughout the study. Similarly, the drug-capping efficiency for LTZ on the nanoparticles demonstrated relative stability. At day 0, the drug-capping efficiency for LTZ was 58.13 ± 2.28%. After 15 days, there had been a slight decrease to 56.65 ± 1.06%, but this variation fell within an acceptable range. By the end of the 30-day period, the drug-capping efficiency for LTZ had increased to 59.54 ± 0.33%. These results suggest that the LTZ capping on the LTZ-BBR@AA-AuNP formulation remained stable or even improved over time. Overall, the stability study indicated consistent colour, transparency, and drug-capping efficiencies for both BBR and LTZ in the LTZ-BBR@AA-AuNP formulation throughout the 30-day period. 

## 3. Discussion

Breast cancer remains one of the deadliest malignancies affecting women worldwide. Aromatase inhibitors, such as LTZ, treat hormone-positive breast cancer in postmenopausal women. The drug has gained popularity because it has an excellent efficacy and safety profile compared to tamoxifen [36]. Like many other drugs, LTZ is poorly soluble in water, is rapidly metabolised, and has several undesirable side effects [37,38]. It is widely known that successful cancer treatment involves the direct administration of particular doses of anticancer medications to cancer cells without damaging nearby normal cells. Using chemotherapeutics and other phytochemicals/naturally occurring bioactive substances is a very modern and innovative approach to cancer therapy [39,40,41,42]. 

An optimal drug delivery system (DDS) capable of conveying many medications and discriminating cancer cells from normal tissues is urgently needed to meet this goal. The current research aims to develop a co-delivery nano system for LTZ and BBR by employing AA-AuNPs as carriers. This system should be able to effectively be internalised into breast cancer cells to have a synergistic impact. The concentrations of LTZ and BBR in the formulation affected the physiological characteristics, including the amounts of entrapped drugs. The experimental findings revealed that the concentrations of LTZ and BBR in AA-AuNP medication were so high that they reached 58 ± 2.9% and 54 ± 2.77%, respectively.

In vitro release research suggested the possibility of developing new LTZ and BBR formulations for 24-h delivery, maybe as injections. LTZ and BBR were shown to have comparable release characteristics, with rapid initial release followed by sustained release over 24 h. The rapid initial release of LTZ and BBR is attributable to drug surface adsorption on the surface or in the outer shell. At a pH of 5.0, it was found that 72.18 ± 3.69% of LTZ, and 68.7 ± 3.43% of BBR had been released after 24 h. At a pH of 7.4, the drug release after 24 h was 64.3 ± 3.215% for LTZ and 56.3 ± 2.815% for BBR.

The results of cell culture experiments indicated that LTZ-BBR@AA-AuNPs were much more cytotoxic than other drugs and free drugs when applied in vitro against MDA-MB-231 cancer cells. The 50% inhibitory concentration (IC_50_) was 2.04 ± 0.011 µg/mL. 

ROS promote cellular damage, apoptotic injuries, and cell death in cell lines [43]. ROS have been implicated in inflammation, ageing, angiogenesis, and cancer conditions. The increased level of ROS in MDA-MB-231 cells may be attributed to a decreased level of estrogen, potentially resulting from the antioxidant effect [44,45,46]. These nanoparticles are reactive to thiols, leading to antioxidant depletion and to cell death by apoptosis in malignant cells. 

Berberine is a plant alkaloid with potent antioxidant and anti-inflammatory activities and is known to promote apoptosis, which is the ultimate objective in cancer treatment [47,48]. Previous research reported its effect on the reduction in cell viability of various cell lines, including MCF-7 and MDA-MB-231 breast cancer cells [49,50]. The results demonstrated an increase in apoptosis compared to the normal control due to increased ROS and enhanced the effect of oncogenes such as caspase 3 and Bax. The flow cytometry results showed that BBR prevented MDA-MB-231 cell growth in phases G1 and S, which is consistent with previous reports [48,49,50]. Previously, combining natural products and LTZ with lower doses highly influenced cell apoptosis by inducing mitochondrial apoptosis [51,52]. 

Overall, the prepared nano formulation seems extremely promising for treating cancers other than breast cancer. Our results suggest that concomitant treatment of LTZ-BBR increases the efficacy of chemotherapeutic agents with low BBR concentrations, especially in chemo-resistant malignancies.

## 4. Materials and Methods

### 4.1. Materials

Letrozole powder (HPLC purity 99.12%) was kindly provided by Hetero drugs Pvt. Ltd., Hyderabad. DMSO (#PHR1309), camptothecin (#C9911), ethanol, berberine chloride, ascorbic acid, and gold chloride (HAuCl_4_.3H_2_O) were purchased from Sigma Aldrich (St. Louis, MO, USA) and used without additional purification. The MDA-MB-231 human breast adenocarcinoma cell line (NCCS, Pune) was used. The cell culture medium—high-glucose DMEM media (Cat. #: AL007)—fetal bovine serum (#RM10432), MTT reagent (5 mg/mL) (# 4060), and D-PBS (#TL1006) were purchased from Himedia, India. A 96-well plate for culturing the cells (from Corning, Bedford, MA, USA) and T25 flask (# 12556009, Biolite-Thermo, Waltham, MA, USA) were also used.

### 4.2. Preparation of Ascorbic Acid Solution (AA) 

The 1% ascorbic acid (AA) stock solution was prepared by dissolving 1 g of AA in a mixture of 50 mL of MilliQ water and 50 mL of ethanol. The solution was shaken at room temperature for 30 min before centrifugation at 5000 rpm. The supernatant obtained was collected and used for the experiment.

### 4.3. Preparation of Gold Chloride Solution

A total of 3.96 mg gold chloride (HAuCl_4_.3H_2_O) was dissolved in 0.5 mL of MilliQ water and 0.5 mL of ethanol to prepare a 10 mM solution.

### 4.4. Synthesis and Optimization of Gold Nanoparticles (AuNPs)

The synthesis of stabilised colloidal AuNPs was performed according to the previous method, with a slide modification in the process [53]. At a temperature of 80 °C ± 1 °C, 100 µL of HAuCl_4_ solution at a concentration of 10 mM was added to 5 mL of AA solution at a concentration of 1 wt% with stirring. After five hours, the previously colourless liquid had changed to a bright ruby red. This confirmed the AuNP synthesis.

### 4.5. Drug Capping

- Capping of BBR and LTZ separately

A solution of 5 mg BBR in 0.5 mL ethanol was prepared. It was added to 5 mL of AA-AuNPs, which were stirred at room temperature for 24 h.

Then, 5 mg of LTZ was mixed with 0.5 mL of ethanol. It was added to 5 mL of AA -AuNPs and kept at room temperature for 24 h with constant stirring. No precipitation of the active ingredient was observed.

- Capping of BBR and LTZ altogether

First, 5 mg of BBR and LTZ were dissolved in 0.5 mL of ethanol and then added to 5 mL of AA-AuNPs with constant stirring at room temperature for 24 h. No precipitation of the drug was observed. The encapsulated drug was measured spectrophotometrically at 240 nm (LTZ) and 422 nm (Shimadzu UV 1601, Japan) (BBR). Equation (1) was used to calculate the fraction of encapsulated drug:

(1) $$\%\;Drug\;capped=\frac{\left(Total\;amount\;of\;LTZ\;and\;BBR\;added-Amount\;of\;free\;LTZ\;and\;BBR\;in\;supernatent\right)}{Total\;amount\;of\;LTZ\;and\;BBR\;added}\times 100$$

### 4.6. Characterization of Nanoparticles

- Hydrodynamic diameter and surface charges measurement

The size was estimated using dynamic light scattering (DLS). The LTZ-BBR@AA-AuNPs’ mean diameter, polydispersity index (PDI) and zeta potential (ZP) were evaluated using a Malvern Zetasizer Nano ZS (Malvern, Model S, Ver. 2.15, UK). The hydrodynamic diameter of the colloidal nanoparticles was evaluated by suspending them in deionised water. DLS was performed using a laser with a wavelength of 633.0 nm, a beam angle of 90°, and a temperature of 25 °C. The results were kept for an average of three cycles and analysed in triplicate for each sample.

- Fourier Transform Infrared analysis

To make a pellet, we combined KBr with various concentrations of ascorbic acid, berberine, letrozole, BBR@AuNPs, LTZ@AuNPs, and LTZ-BBR@AA-AuNPs. The pellets were analysed using an FTIR spectrophotometer (Spectrum BX, Perkin Elmer, Beaconsfield, UK) working in the 400–4000 cm^−1^ wavenumber range.

### 4.7. Morphology Study

The shape of the LTZ-BBR@AA-AuNPs was investigated by high-resolution transmission electron microscopy (HR-TEM, JEOL 2100 HRTEM, Republic of Korea). To capture a sample drop, a copper grid that had been coated with carbon was used. A 2% uranyl acetate stain was applied after air drying at room temperature.

### 4.8. In Vitro Drug Release Rtudies

Dialysis bag dissolutions were conducted at 37 °C in phosphate-buffered saline (PBS, pH 7.4) and sodium acetate buffer (pH 5.0) to determine the in vitro release of LTZ-BBR from LTZ-BBR@AA-AuNPs. The dialysis membrane had a molecular weight cutoff (MWCO) of 12000 Da. In addition, 500 mL of release media was prepared using pH 7.4 and pH 5.0 buffer solutions. At predefined intervals during the course of 24 h, 2 mL samples were collected and then diluted with fresh medium. A UV-Vis spectrophotometer was used to measure both LTZ (240 nm) and BBR (422 nm) emissions.

### 4.9. Anticancer Activity of Test Compounds In Vitro by MTT Assay

The MDA-MB-231 TNBC cell line is a good example of the epithelial–mesenchymal transition (EMT) linked to breast cancer metastasis [54]. Cell viability in the MDA-MB-231 cell line was evaluated using the MTT assay of LTZ-BBR@AA-AuNPs. Cells were subcultured every two days in a 5% CO_2_ and 18–20% O_2_ atmosphere at 37 °C in a CO_2_ incubator with a DMEM medium containing high glucose content, 10% FBS, and 1% antibiotic solution. Without test media, 200 μL of cell suspension was seeded on a 96-well plate at the desired cell density (20,000 cells per well). After 24 h of culture, the corresponding test materials were added at the indicated concentrations. The plates were kept in an incubator for 48 h at 37 °C with 5% CO_2_. After the incubation time, the plates were removed from the incubator, and MTT reagent was introduced to them. The plates were wrapped in aluminium foil to prevent exposure to light. Again, they were placed in the incubator for 3 h. The MTT reagent was then discarded. Following that, 100 µL of DMSO was added to the mixture. To enhance dissolution, a gyro-shaker was used to provide gentle agitation. We used a 570 nm wavelength spectrophotometer to measure the absorbance. The percentage of viable cells can be determined using the following formula:

(2) $$\%\;Cell\;viability=\frac{\left(A570\;nm\;treated\;cells\right)}{\left(A570\;nm\;untreated\;cells\right)}\times 100$$

The IC_50_ value was determined using a linear regression equation, i.e., y = mx + c. Here, y = 50, and the m and c values were derived from the viability graph.

### 4.10. Estimation of Intracellular Reactive Oxygen Species (ROS)

The MDA-MB-231 cell line was seeded in DMEM supplemented with 10% *v*/*v* FBS and 1% penicillin/streptomycin in 96-well plates at a density of 1.2 × 10^4^ (37 °C, 5% CO_2_) in order to determine the amount of intracellular ROS using the enzyme-linked immunosorbent assay (ELISA). After removing the medium, 0.1 mL/well of each of the treatments and reference solutions was applied to each well of the experiment (*n* = 4). The treatments (naked LTZ, naked BBR, LTZ@AuNPs, BBR@AuNPs, and their LTZ-BBR combination LTZ-BBR@AA-AuNPs) dispersed in DMEM were added at a specified concentration (50% of the specified IC_50_ in the cytotoxicity study mentioned above) and left for 48 h, while the control wells were untreated. This was performed so that the results could be compared with those of the cytotoxicity study. The ROS ELISA assay was estimated according to the method given by Mekkawy et al. [55]. Using a calibration curve made from standard solutions, we were able to calculate the ROS level by measuring the absorbance at 450 nm and comparing it with that of the control and samples we examined.

### 4.11. Cell Cycle Analysis by Flow Cytometry

Apoptosis was quantitatively analysed by flow cytometry after cells were stained with annexin V and propidium iodide (PI). In 6-well plates, MDA-MB-231 cells were seeded at a density of 1 × 10^5^ cells per well, and the plates were left to incubate overnight. After that, the cells were placed into a 24 h incubator while being treated with DMSO, LTZ, BBR, LTZ@AuNPs, BBR@AuNPs, and LTZ-BBR@AA-AuNPs. In the cytotoxicity study, treatments were applied at 50% of the indicated concentration of IC_50_. The cells were then rinsed, trypsinised, and washed with DMEM media; they were captured in falcon tubes in which the corresponding supernatant was obtained, and the cell pellets were washed two times with PBS. Subsequently, 100 µL of cell suspension was transferred to a FACS tube and combined with 5 µL of annexin V-FITC and 5 µL of PI. The tubes were slightly yet thoroughly mixed before being incubated for 30 min at room temperature in the dark. Subsequently, within 1 h, 400 µL of the binding buffer had been added to the FACS tube, and the tube was processed through the FACS machine.

### 4.12. Stability Studies

Short-term physical stability was evaluated to determine how temperature affects LTZ-BBR@AA-AuNPs. The 30-day stability of LTZ-BBR@AA-AuNPs was evaluated at a temperature of 25 °C. The physical stability of LTZ-BBR@AA-AuNPs was evaluated by measuring the colour, encapsulation, and transparency.

## 5. Conclusions

There is an evident enthusiasm among researchers to develop a unified platform that combines nanoagents and nanocarriers, aiming to gain a comprehensive understanding of the bioavailability and effectiveness of molecular-based anticancer agents. The objective goes beyond creating mere new treatments, focusing on novel strategies capable of overcoming the resistance encountered with traditional chemotherapy approaches. A promising illustration of this approach is the successful fabrication of LTZ-BBR@AA-AuNPs for breast cancer treatment. By encapsulating anticancer drugs or herbal compounds within nanoparticles, several advantageous outcomes have been observed, including improved drug release, enhanced penetration into cancer cells, and facilitated endocytosis. These mechanisms enable a synergistic approach to cancer treatment, promoting higher efficacy in patient recovery while concurrently monitoring the potential toxicity associated with the administration of natural or synthetic anticancer drugs. Collectively, it is evident that the medical community persists in its quest to develop increasingly sophisticated methodologies in the battle against one of the most devastating diseases affecting humanity.

## Figures and Tables

**Table 1:** Stability study of LTZ-BBR@AA-AuNPs.

Days	Colour	Transparency	Drug Capping @BBR	Drug Capping @LTZ
0	Yellow	Transparent	54.33 ± 0.47	58.13 ± 2.28
15	Yellow	Transparent	54.87 ± 0.17	56.65 ± 1.06
30	Yellow	Transparent	56.14 ± 0.65	59.54 ± 0.33

## Data Availability

Data is contained within the article.

## References

[1] Aldawsari H.M., Singh S., Alhakamy N.A., Bakhaidar R.B., Halwani A.A., Badr-Eldin S.M. (2021). Gum Acacia Functionalized Colloidal Gold Nanoparticles of Letrozole as Biocompatible Drug Delivery Carrier for Treatment of Breast Cancer. Pharmaceutics.

[2] Bhushan A., Gonsalves A., Menon J.U. (2021). Current state of breast cancer diagnosis, treatment, and theranostics. Pharmaceutics.

[3] Sung H., Ferlay J., Siegel R.L., Laversanne M., Soerjomataram I., Jemal A., Bray F. (2021). Global Cancer Statistics 2020: GLOBOCAN Estimates of Incidence and Mortality Worldwide for 36 Cancers in 185 Countries. CA Cancer J. Clin..

[4] Hooshyar S.P., Panahi H.A., Moniri E., Farsadrooh M. (2021). Tailoring a new hyperbranched PEGylated dendrimer nano-polymer as a super-adsorbent for magnetic solid-phase extraction and determination of letrozole in biological and pharmaceutical samples. J. Mol. Liq..

[5] Rubovszky G., Kocsis J., Boér K., Chilingirova N., Dank M., Kahán Z., Kaidarova D., Kövér E., Krakovská B.V., Máhr K. (2022). Systemic Treatment of Breast Cancer. 1st Central-Eastern European Professional Consensus Statement on Breast Cancer. Pathol. Oncol. Res..

[6] Mátrai Z., Kelemen P., Kósa C., Maráz R., Paszt A., Pavlovics G., Sávolt Á., Simonka Z., Tóth D., Kásler M. (2022). Modern Breast Cancer Surgery 1st Central-Eastern European Professional Consensus Statement on Breast Cancer. Pathol. Oncol. Res..

[7] Costa B., Amorim I., Gärtner F., Vale N. (2020). Understanding Breast cancer: From conventional therapies to repurposed drugs. Eur. J. Pharm. Sci..

[8] Li L., Xu X., Fang L., Liu Y., Sun Y., Wang M., Zhao N., He Z. (2010). The transdermal patches for site-specific delivery of letrozole: A new option for breast cancer therapy. AAPS PharmSciTech.

[9] McDonnell D.P., Wardell S.E., Norris J.D. (2015). Oral Selective Estrogen Receptor Downregulators (SERDs), a Breakthrough Endocrine Therapy for Breast Cancer. J. Med. Chem..

[10] Nair H.B., Huffman S., Veerapaneni P., Kirma N.B., Binkley P., Perla R.P., Evans D.B., Tekmal R.R. (2011). Hyaluronic acid-bound letrozole nanoparticles restore sensitivity to letrozole-resistant xenograft tumors in mice. J. Nanosci. Nanotechnol..

[11] Shaban M., Ghaffary S., Hanaee J., Karbakhshzadeh A., Soltani S. (2021). Synthesis and characterization of new surface modified magnetic nanoparticles and application for the extraction of letrozole from human plasma and analysis with HPLC-fluorescence. J. Pharm. Biomed. Anal..

[12] Undevia S.D., Gomez-Abuin G., Ratain M.J. (2005). Pharmacokinetic variability of anticancer agents. Nat. Rev. Cancer.

[13] Akbarzadeh I., Saremi Poor A., Khodarahmi M., Abdihaji M., Moammeri A., Jafari S., Salehi Moghaddam Z., Seif M., Moghtaderi M., Lalami Z.A. (2022). Gingerol/letrozole-loaded mesoporous silica nanoparticles for breast cancer therapy: In-silico and in-vitro studies. Microporous Mesoporous Mater..

[14] Housman G., Byler S., Heerboth S., Lapinska K., Longacre M., Snyder N., Sarkar S. (2014). Drug resistance in cancer: An overview. Cancers.

[15] Jia J., Zhu F., Ma X., Cao Z.W., Li Y.X., Chen Y.Z. (2009). Mechanisms of drug combinations: Interaction and network perspectives. Nat. Rev. Drug Discov..

[16] Cheng F., Kovács I.A., Barabási A.L. (2019). Network-based prediction of drug combinations. Nat. Commun..

[17] Babaei M., Evers T.M.J., Shokri F., Altucci L., de Lange E.C.M., Mashaghi A. (2023). Biochemical reaction network topology defines dose-dependent Drug–Drug interactions. Comput. Biol. Med..

[18] Bagga A.S., Sanzgiry S.M. (1982). Chemotherapy of pulmonary tuberculosis. Tuberc. Respir. Dis..

[19] Yang A.-K., He S.-M., Liu L., Liu J.-P., Qian Wei M., Zhou S.-F. (2010). Herbal Interactions with Anticancer Drugs: Mechanistic and Clinical Considerations. Curr. Med. Chem..

[20] Atallah M.A., Sallam M.A., Abdelmoneem M.A., Teleb M., Elkhodairy K.A., Bekhit A.A., Khafaga A.F., Noreldin A.E., Elzoghby A.O., Khattab S.N. (2022). Green self-assembled lactoferrin carboxymethyl cellulose nanogels for synergistic chemo/herbal breast cancer therapy. Colloids Surf. B Biointerfaces.

[21] Khan I., Joshi G., Nakhate K.T., Ajazuddin, Kumar R., Gupta U. (2019). Nano-Co-Delivery of Berberine and Anticancer Drug Using PLGA Nanoparticles: Exploration of Better Anticancer Activity and In Vivo Kinetics. Pharm. Res..

[22] Yin S.Y., Wei W.C., Jian F.Y., Yang N.S. (2013). Therapeutic applications of herbal medicines for cancer patients. Evid. -Based Complement. Altern. Med..

[23] Bazrafshani M.S., Khandani B.K., Pardakhty A., Tajadini H., Pour Afshar R.M., Moazed V., Nemati A., Nasiri N., Sharifi H. (2019). The prevalence and predictors of using herbal medicines among Iranian cancer patients. Complement. Ther. Clin. Pract..

[24] Park J., Jeong D., Song M., Kim B. (2021). Recent advances in anti-metastatic approaches of herbal medicines in 5 major cancers: From traditional medicine to modern drug discovery. Antioxidants.

[25] Vemuri S.K., Banala R.R., Mukherjee S., Uppula P., GPV S., Gurava G.R., Malarvilli T. (2019). Novel biosynthesized gold nanoparticles as anti-cancer agents against breast cancer: Synthesis, biological evaluation, molecular modelling studies. Mater. Sci. Eng. C.

[26] Li N., Shang Y., Han Z., Wang T., Wang Z.G., Ding B. (2019). Fabrication of Metal Nanostructures on DNA Templates. ACS Appl. Mater. Interfaces.

[27] Devi L., Gupta R., Jain S.K., Singh S., Kesharwani P. (2020). Synthesis, characterization and in vitro assessment of colloidal gold nanoparticles of Gemcitabine with natural polysaccharides for treatment of breast cancer. J. Drug Deliv. Sci. Technol..

[28] Kong T., Zeng J., Wang X., Yang X., Yang J., McQuarrie S., McEwan A., Roa W., Chen J., Xing J.Z. (2008). Enhancement of radiation cytotoxicity in breast-cancer cells by localized attachment of gold nanoparticles. Small.

[29] Ghosn Y., Kamareddine M.H., Tawk A., Elia C., El Mahmoud A., Terro K., El Harake N., El-Baba B., Makdessi J., Farhat S. (2019). Inorganic Nanoparticles as Drug Delivery Systems and Their Potential Role in the Treatment of Chronic Myelogenous Leukaemia. Technol. Cancer Res. Treat..

[30] Lazarus G.G., Singh M. (2016). In vitro cytotoxic activity and transfection efficiency of polyethyleneimine functionalized gold nanoparticles. Colloids Surf. B Biointerfaces.

[31] Jang K.-I., Hyeon G.L. (2008). Stability of chitosan nanoparticles for L-ascorbic acid during heat treatment in aqueous solution. J. Agric. Food Chem..

[32] Yin X., Chen K., Cheng H., Chen X., Feng S., Song Y., Liang L. (2022). Chemical Stability of Ascorbic Acid Integrated into Commercial Products: A Review on Bioactivity and Delivery Technology. Antioxidants.

[33] Murphy M.P., Bayir H., Belousov V., Chang C.J., Davies K.J.A., Davies M.J., Dick T.P., Finkel T., Forman H.J., Janssen-Heininger Y. (2022). Guidelines for measuring reactive oxygen species and oxidative damage in cells and in vivo. Nat. Metab..

[34] Prieto-Bermejo R., Romo-González M., Pérez-Fernández A., Ijurko C., Hernández-Hernández Á. (2018). Reactive oxygen species in haematopoiesis: Leukaemic cells take a walk on the wild side. J. Exp. Clin. Cancer Res..

[35] Perillo B., Di Donato M., Pezone A., Di Zazzo E., Giovannelli P., Galasso G., Castoria G., Migliaccio A. (2020). ROS in cancer therapy: The bright side of the moon. Exp. Mol. Med..

[36] Alemrayat B., Elhissi A., Younes H.M. (2019). Preparation and characterization of letrozole-loaded poly(d,l-lactide) nanoparticles for drug delivery in breast cancer therapy. Pharm. Dev. Technol..

[37] Coates A.S., Keshaviah A., Thürlimann B., Mouridsen H., Mauriac L., Forbes J.F., Paridaens R., Castiglione-Gertsch M., Gelber R.D., Colleoni M. (2007). Five years of letrozole compared with tamoxifen as initial adjuvant therapy for postmenopausal women with endocrine-responsive early breast cancer: Update of study BIG 1-98. J. Clin. Oncol..

[38] Alemrayat B., Elhissi A., Younes H. (2019). Preparation and Characterization of Letrozole-Loaded Poly (D,L-Lactide) Nanoparticles for Breast Cancer Therapy. Qatar Foundation Annual Research Conference Proceedings.

[39] Muhamad N., Plengsuriyakarn T., Na-Bangchang K. (2018). Application of active targeting nanoparticle delivery system for chemotherapeutic drugs and traditional/herbal medicines in cancer therapy: A systematic review. Int. J. Nanomed..

[40] Abdulridha M.K., Al-Marzoqi A.H., Al-awsi G.R.L., Mubarak S.M.H., Heidarifard M., Ghasemian A. (2020). Anticancer Effects of Herbal Medicine Compounds and Novel Formulations: A Literature Review. J. Gastrointest. Cancer.

[41] Majidzadeh H., Araj-Khodaei M., Ghaffari M., Torbati M., Ezzati Nazhad Dolatabadi J., Hamblin M.R. (2020). Nano-based delivery systems for berberine: A modern anti-cancer herbal medicine. Colloids Surf. B Biointerfaces.

[42] Mughees M., Wajid S. (2020). Herbal Based Polymeric Nanoparticles as a Therapeutic Remedy for Breast Cancer. Anticancer Agents Med. Chem..

[43] Gibellini L., Pinti M., Nasi M., de Biasi S., Roat E., Bertoncelli L., Cossarizza A. (2010). Interfering with ROS metabolism in cancer cells: The potential role of quercetin. Cancers.

[44] Agarwal A., Doshi S. (2013). The role of oxidative stress in menopause. J. Midlife Health.

[45] Bourgonje A.R., Abdulle A.E., Al-Rawas A.M., Al-Maqbali M., Al-Saleh M., Enriquez M.B., Al-Siyabi S., Al-Hashmi K., Al-Lawati I., Bulthuis M.L.C. (2020). Systemic oxidative stress is increased in postmenopausal women and independently associates with homocysteine levels. Int. J. Mol. Sci..

[46] Peng B., Zhang S.-Y., Chan K.I., Zhong Z.-F., Wang Y.-T. (2023). Novel Anti-Cancer Products Targeting AMPK: Natural Herbal Medicine against Breast Cancer. Molecules.

[47] Kong W., Wei J., Abidi P., Lin M., Inaba S., Li C., Wang Y., Wang Z., Si S., Pan H. (2004). Berberine is a novel cholesterol-lowering drug working through a unique mechanism distinct from statins. Nat. Med..

[48] Wang K., Feng X., Chai L., Cao S., Qiu F. (2017). The metabolism of berberine and its contribution to the pharmacological effects. Drug Metab. Rev..

[49] Wang Z.C., Wang J., Chen H., Tang J., Bian A.W., Liu T., Yu L.F., Yi Z., Yang F. (2020). Synthesis and anticancer activity of novel 9,13-disubstituted berberine derivatives. Bioorganic Med. Chem. Lett..

[50] Rauf A., Abu-Izneid T., Khalil A.A., Imran M., Shah Z.A., Bin Emran T., Mitra S., Khan Z., Alhumaydhi F.A., Aljohani A.S.M. (2021). Berberine as a potential anticancer agent: A comprehensive review. Molecules.

[51] Chiu C.F., Fu R.H., Hsu S.H., Yu Y.H., Yang S.F., Tsao T.C.Y., Chang K.B., Yeh C.A., Tang C.M., Huang S.C. (2021). Delivery capacity and anticancer ability of the berberine-loaded gold nanoparticles to promote the apoptosis effect in breast cancer. Cancers.

[52] Javed Iqbal M., Quispe C., Javed Z., Sadia H., Qadri Q.R., Raza S., Salehi B., Cruz-Martins N., Abdulwanis Mohamed Z., Sani Jaafaru M. (2021). Nanotechnology-Based Strategies for Berberine Delivery System in Cancer Treatment: Pulling Strings to Keep Berberine in Power. Front. Mol. Biosci..

[53] Alqarni M.H., Foudah A.I., Alam A., Salkini M.A., Muharram M.M., Labrou N.E., Kumar P. (2022). Development of Gum-Acacia-Stabilized Silver Nanoparticles Gel of Rutin against Candida albicans. Gels.

[54] Huang Z., Yu P., Tang J. (2020). Characterization of triple-negative breast cancer MDA-MB-231 cell spheroid model. Onco Targets Ther..

[55] Mekkawy A.I., Eleraky N.E., Soliman G.M., Elnaggar M.G., Elnaggar M.G. (2022). Combinatorial Therapy of Letrozole- and Quercetin-Loaded Spanlastics for Enhanced Cytotoxicity against MCF-7 Breast Cancer Cells. Pharmaceutics.

